# The clinical significance of incidental intra-abdominal findings on positron emission tomography performed to investigate pulmonary nodules

**DOI:** 10.1186/1477-7819-10-25

**Published:** 2012-01-27

**Authors:** Richdeep S Gill, Troy Perry, Jonathan T Abele, Eric LR Bédard, Daniel Schiller

**Affiliations:** 1 Department of Surgery, University of Alberta, Edmonton, Alberta, Canada; 2 Department of Radiology and Diagnostic Imaging, University of Alberta, Edmonton, Alberta, Canada; 3 Divisions of Thoracic and General Surgery, Department of Surgery, University of Alberta, Edmonton, Canada

**Keywords:** Pulmonary nodules, incidental intraabdominal findings, PET scan, lung cancer, colon cancer, adenoma

## Abstract

**Background:**

Lung cancer is a common cause of cancer-related death. Staging typically includes positron emission tomography (PET) scanning, in which^18^F-fluoro-2-dexoy-D-glucose (FDG) is taken up by cells proportional to metabolic activity, thus aiding in differentiating benign and malignant pulmonary nodules. Uptake of FDG can also occur in the abdomen. The clinical significance of incidental intraabdominal FDG uptake in the setting of pulmonary nodules is not well established. Our objective was to report on the clinical significance of incidental intra-abdominal FDG activity in the setting of lung cancer.

**Methods:**

Fifteen hundred FDG-PET reports for studies performed for lung cancer were retrospectively reviewed for the presence of incidental FDG-positive intraabdominal findings. Patient charts with positive findings were then reviewed and information extracted.

**Results:**

Twenty-five patients (25/1500) demonstrated incidental intraabdominal FDG uptake thought to be significant (1.7%) with a mean patient age of 71 years. Colonic uptake was most common (n = 17) with 9 (52%) being investigated further. Of these 9 cases, a diagnosis of malignancy was made in 3 patients, pre-malignant adenomas in 2 patients, a benign lipoma in 1 patient and no abnormal findings in the remaining patients. 8 patients were not investigated further (3 diagnosed with metastatic lung cancer and 2 were of advanced age) secondary to poor prognosis.

**Conclusion:**

Incidental abdominal findings in the colon on FDG-PET scan for work-up of pulmonary nodules need to be further investigated by colonoscopy.

## Background

**Figure 1:**
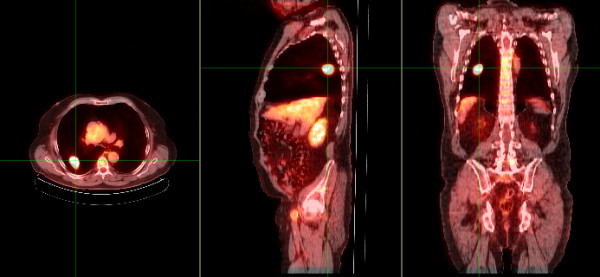
**Fused coronal, sagittal and transaxial FDG PET/CT images in an 84 year old male (Patient 11)**. The cross-hairs localize an FDG-avid lung cancer.

Lung cancer is the most common cause of cancer-related deaths worldwide. Staging typically uses imaging techniques such as chest radiography, computed tomography (CT), and occasionally magnetic resonance imaging (MRI). In the last decade, use of positron emission tomography (PET) has been increasingly employed to improve both the staging of lung cancer and the assessment of patients with pulmonary nodules (Figure 1) [1-3]. Cells in a malignant tumor undergo glycolysis at an increased rate, and hence have greater cellular uptake of glucose [4]. ^18^F-fluoro-2-dexoy-D-glucose (FDG) is a glucose analog that undergoes the same mechanism of uptake as glucose but becomes trapped within the tumor cell. FDG emits a positron, which can ultimately be imaged making it a good tracer for metabolic activity [5,6]. Uptake of FDG can also occur in other cells within the body, leading to incidental extra-thoracic findings in this setting [1]. Incidental foci of FDG uptake may include striated muscle, inflammation, thrombosis and second primary malignancies [1,7,8]. These incidental primary malignancies may include colon cancer, which has risk factors in common with lung cancer, namely advanced age and smoking [9].

Previous studies have assessed the efficacy of PET for lung cancer staging and have reported incidental extra-thoracic findings. The clinical significance of incidental FDG uptake within the abdomen in asymptomatic patients remains unclear. Our objective is to review the experience at our institution and to evaluate the clinical significance of incidental intra-abdominal findings on FDG-PET scans performed for staging of lung cancer or the assessment of pulmonary nodules.

## Methods

Ethics approval was obtained from the Health Research Ethics Board at the University of Alberta prior to commencement of this project.

### Patient Population

A report database of FDG-PET scans performed between October 2002 and April 2008 at the major regional cancer center (Cross Cancer Institute, Edmonton, Alberta), was initially filtered according to indication and we selected out scans performed for 'investigation of pulmonary nodules'. This captured 1500 FDG-PET scan reports performed for evaluation of pulmonary nodules as part of staging for lung cancer or assessment of malignant potential. The 1500 FDG-PET scans were then reviewed for presence of incidental FDG-positive intraabdominal findings. Incidental intraabdominal FDG-uptake was defined as "focal" and intense uptake compared to background activity as observed by the nuclear medicine physicians. There was no specific maximum standardized uptake value (SUVmax) to define incidental focal uptake. However, a number of experienced nuclear medicine physicians interpreted the FDG-PET scans and based on their interpretation reported significant intraabdominal FDG-uptake. PET imaging reports indicating focal FDG-uptake in the abdomen were assumed to be abnormal. Patients with prior computed tomographic (CT) or magnetic resonance imaging indicating extra-thoracic disease were excluded. Also, patients with known diagnoses of intra-abdominal malignancy were excluded. Patient charts with incidental intraabdominal findings were then retrospectively reviewed and the following data was extracted: patient information including demographics, risk factors, type and clinical stage of lung cancer, and treatment. In patients with FDG-positive lung and abdominal findings, information on investigations of incidental abdominal findings, diagnosis and treatment were extracted.

### FDG-PET Protocol

At our Regional Cancer Center (standard whole body FDG protocols from the skull base to the proximal thighs were used. PET scans were obtained using a dedicated PET (Philips Allegro, Best, the Netherlands) or PET/CT scanner (Philips Gemini, Best, the Netherlands). Patients fasted for a minimum of 6 h before FDG injection. FDG was administered at a dose of 5.2 MBq/kg ± 10% intravenously with a maximum dose of 740 MBq. PET images were acquired at 3 to 4 minutes per bed position. For PET studies attenuation correction was performed using a^137^Cs transmission source. For PET/CT studies, CT attenuation correction was applied. Images were reconstructed using an ordered-subset expectation maximization iterative reconstruction algorithm.

## Results

**Figure 2:**
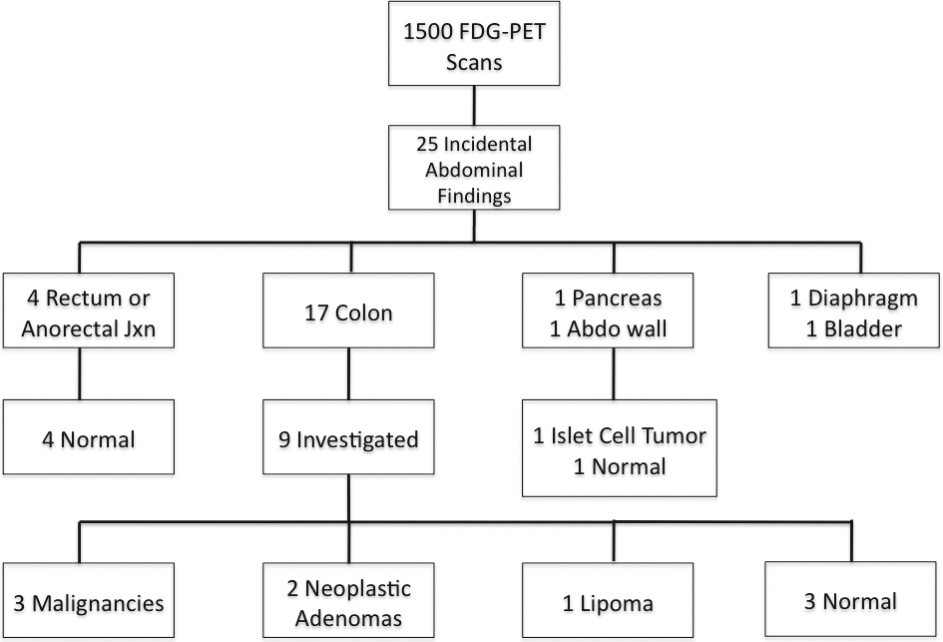
**Flow diagram of reviewed FDG-PET scans (Abdo = abdominal; jxn = junction)**.

**Figure 3:**
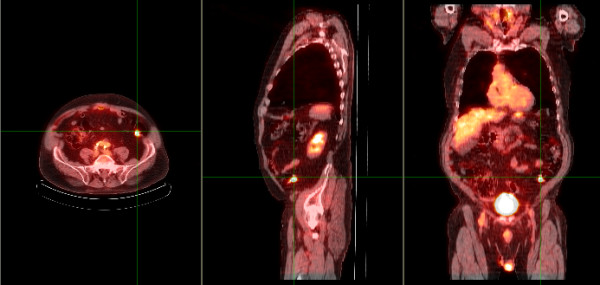
**Fused coronal, sagittal and transaxial FDG PET/CT images in an 84 year old patient (Patient 11)**. The cross-hairs localize intense uptake in the left colon.

A total of 1500 FDG-PET scans were reviewed, with 25 demonstrating incidental intraabdominal findings (1.7%) (Figure 2). As seen in Table 1, the mean age of patients with incidental intraabdominal findings was 71 years (range: 30 to 89 years of age), with 52% females. The most common location within the abdomen for focal FDG uptake was the colon (n = 17), followed by the rectal or anorectal junction (n = 4). Of the 17 cases with incidental colonic findings, 9 (52%) were investigated further with either contrast-enhanced CT abdominal scan or colonoscopy. None of the four cases with rectal/anorectal findings were investigated. Of the nine colonic cases, a diagnosis of malignancy was made in three patients, pre-malignant adenomas in two patients (Figure 3), a benign lipoma in one patient and no abnormal findings in the remaining patients (Table 2). Of the eight patients with focal colonic FDG uptake not investigated further, three patients (patients 3, 4, and 6) had metastatic lung cancer and two patients (patients 14 and 15) were of advanced age. For the other three patients with focal colonic FDG uptake, chart review indicates that no further investigations were performed. Further details of investigations performed for colonic incidental findings are given in Table 2.

**Table 1:** Demographics and FDG-PET Scan Findings

			PET Scan					
**Pt**	**Age (yrs)**	**Sex**	**Lung FDG+**	**ABDO FDG+**	**ABDO FDG+ PET site**	**Pulmonary Diagnosis**	**ABDO Findings Invest**	**Abdominal Diagnosis**

1	71	M	+	+	Colon	N/A	Yes	Lipoma

2	64	M	+	+	Colon	NSCLC	Yes	Normal

3	79	M	+	+	Colon	Metastatic NSCLC	No	-

4	63	F	+	+	Colon	Metastatic NSCLC	No	-

5	84	F	+	+	Right Colon	Adenocarcinoma	No	-

6	81	F	+	+	Right Colon	Metastatic NSCLC	No	-

7	64	F	+	+	Right Colon	Bronchoalveolar cancer	Yes	Normal

8	67	F	+	+	Right colon	SCC	Yes	Normal

9	74	F	+	+	Transverse Colon	Metastasis from Colon Cancer	Yes	Colon Adenocarcinoma

10	82	M	+	+	Left colon	Bronchogenic cancer	Yes	Colon Adenocarcinoma

11*	84	M	+	+	Left Colon	NSCLC	Yes	Colon Adenocarcinoma

12	80	M	-	+	Sigmoid Colon	Benign	No	-

13	75	M	+	+	Sigmoid Colon	Benign	Yes	Adenoma

14	89	F	+	+	Sigmoid Colon	Bronchogenic cancer	No	-

15	88	F	-	+	Sigmoid Colon	Benign	No	-

16	77	M	+	+	Sigmoid Colon	SCC	No	-

17	77	M	-	+	Rectosigmoid	Benign	Yes	Adenoma

18	30	M	+	+	Rectum	Reactive Lymphoid Hyperplasia	No	-

19	54	M	+	+	Anorectal junction	Spindle cell Cancer	No	-

20	78	F	+	+	Anorectal junction	NSCLC	No	-

21*	64	F	+	+	Anorectal junction	NSCLC	No	-

22	65	F	+	+	Diaphragm	Adenocarcinoma	No	-

23	74	F	+	+	Bladder	NSCLC	No	-

24	54	M	+	+	Pancreas	Benign	Yes	Islet Cell Tumor

25	49	F	+	+	ABDO wall	N/A	Yes	Normal

**Mean**	**71**	**52% F**						

M = male; F = female; + = positive; - = negative; N/A = not available; ABDO = abdominal; FDG = flourodeoxyglucose; SCC = squamouse cell carcinoma; NSCLC = non-small cell lung cancer; * PET-CT Imaging performed

**Table 2:** Diagnosis and Treatment of Incidental Colonic Findings

Pt	ABDO FDG+ PET site	SUVmax	Investigations	Intraabdominal Diagnosis	Treatment	Final Pathology
1	Colon	4.1	Colonoscopy	Lipoma in colon	Endoscopic Polypectomy	Lipoma

2	Colon	5.7	Colonoscopy	Normal	-	Normal

7	Right Colon	3.8	Colonoscopy	Normal	-	Normal

8	Right colon	9.5	CT Abdomen	Normal	-	Normal

9	Transverse Colon	5.3	CT Abdomen	Metastatic Colon Cancer	Right Hemicolectomy	Metastatic Colon Adenocarcinoma

10	Left colon	6.1	Colonoscopy	Villous Adenoma	Left Hemicolectomy	Colon Adenocarcinoma

11	Left Colon	8.8	Colonoscopy	Colon Cancer	Endoscopic Polypectomy	Colon Adenocarcinoma

13	Sigmoid Colon	N/A	Colonoscopy	Tubulovillous Adenoma	Endoscopic Polypectomy	Adenoma

17	Rectosigmoid Colon	12.8	Endoscopy	Villous Adenoma	Endoscopic Polypectomy	Adenoma

24	Pancreas	6.1	CT Abdomen	Endocrine tumor	Whipple's Procedure	Islet Cell Tumor

25	ABDO wall	1.5	US Abdomen	Normal	-	Normal

ABDO = abdominal; FDG = fluorodeoxyglucose; + = positive; US = ultrasound; CT = computed tomography; SUVmax = maximum standardized uptake value; N/A = not available

A patient with incidental FDG uptake in the pancreas was diagnosed with an insulinoma and received surgical resection by pancreaticoduodenectomy. A patient with incidental FDG uptake in the abdominal wall, was found to have no remarkable findings on abdominal ultrasound. Two patients with FDG uptake in the diaphragm and bladder were not investigated further according to medical records.

## Discussion

Our retrospective review demonstrates that incidental FDG positive intraabdominal findings on PET imaging for lung cancer may represent clinically significant pathology, especially if the lesion is located in the colon. Of the nine patients investigated for incidental FDG uptake within the colon, three (33%) were ultimately found to have colon cancer, with two others (22%) having pre-malignant lesions (adenomas). Pre-malignant lesions such as colonic adenomas have been previously reported to accumulate FDG on PET imaging [10].

Zhuang et al retrospectively assessed the records of 500 patients evaluated by FDG-PET imaging for pulmonary nodules at their institution and found that 17 had colonic uptake with 5 (35%) proven colon cancers [11]. Beatty et al reported similar findings, in which PET/CT imaging was performed on over 2000 cancer patients [12]. Of the 133 patients investigated for incidental findings on PET/CT imaging, 31% were found to have a second primary malignancy [12]. Interestingly, PET imaging has been shown to be highly sensitive for intra-luminal colon cancer. In a study by Abdel-Nabi et al, PET imaging assessed 37 patients with known colon cancer and all carcinomas were identified (sensitivity, 100%) [13]. These authors estimated a positive predictive value of 90% [13]. Contrastingly, Weston et al reported a positive predictive value closer to 65% [14]. This discrepancy may be related to the sensitivity of PET imaging being influenced by both size and histology of the colonic lesion [14].

One of the advantages of staging cancer patients with PET imaging is the ability to detect not only occult metastatic disease but also synchronous malignancies. Age remains the most significant risk factor for colorectal cancer [15], and as seen in our review, most patients with lung cancer were of advanced age. The most significant risk factor for lung cancer remains smoking. According to a review by Giovannucci, tobacco is strongly associated with colon cancer and tobacco use is associated with a 2-3 fold increased risk of colorectal adenoma. These authors speculate that smoking may be the potential source of up to one in five colorectal cancers [9].

There are several limitations to this study. The main limitation of this study is the retrospective nature. Second, of the 1500 PET scans performed at our institution, we were unable to filter the results to isolate those performed for lung cancer staging and those for investigation of pulmonary nodules. Third, there were a number of patients either lost to follow-up or who may have been investigated without appropriate documentation. These factors can lead to sample bias, however correlation of our results with other studies is supportive. Furthermore, since only PET scans with positive FDG uptake were reviewed further; we cannot comment on false-negative rates of PET scan. One final limitation of this study is that there were a mixture of PET only and PET/CT imaging studies performed. The CT component may provide additional information potentially improving the anatomic localization and specificity of the PET findings, although this is controversial [16]. Modern standard-of-care is to perform PET/CT for all patients being investigated for pulmonary nodules or being staged for lung cancer.

As lung cancer continues to be the most common cause of cancer-related death in North America, PET imaging will be increasingly used to evaluate pulmonary nodules. The frequency of incidental findings of focal hypermetabolism can be expected to increase and clinicians will be faced with the decision of how best to investigate these patients, if at all. With the majority of these findings representing malignant or premalignant neoplasm [17], they can be classified as clinically significant despite lack of symptomatology. Our study found that the majority (56%) of cases with incidental colonic FDG uptake had an underlying diagnosis of malignant or premalignant lesions in the colon. This is contrary to our findings of FDG uptake in the head and neck region, in which there was a low incidence of malignancy (unpublished data). Evaluation of incidental focal colonic FDG uptake with colonoscopy and biopsy is recommended, as colonic pathology is often undetectable by ultrasound, CT scanning and MRI. Review of the PET imaging with the endoscopist may also lead to better localization of the lesion [17]. Treatment plans will continue to be individualized based on the primary pulmonary diagnosis. Further prospective studies are needed to define clear criteria for further investigation of potentially significant incidental intraabdominal FDG-positive findings.

## Conclusion

In conclusion, FDG-positive incidental intraabdominal findings on PET imaging during work-up of pulmonary nodules, may represent clinically significant pathology, especially if localized to the colon. Further investigation with colonoscopy is recommended.

## Competing interests

The authors declare that they have no competing interests.

## Authors' contributions

RSG: Participated in study design, data collection and analysis. Writing manuscript and revisions. Approved final version of manuscript.

TP: Participated in study design and data collection. Writing manuscript and revisions. Approved final version of manuscript.

JTA: Creation of figures. Writing and revising manuscript. Approved final version of manuscript.

ELRB: Study design and analysis. Revising manuscript. Approved final version of manuscript.

DS: Study design and analysis. Revising manuscript. Approved final version of manuscript.
